# Kardiovaskuläre Prävention in Sachsen-Anhalt: Notwendigkeit und neue Perspektiven

**DOI:** 10.1007/s00108-024-01789-x

**Published:** 2024-10-10

**Authors:** Patrick Müller, Maximilian Herzog, Yves Duderstadt, Matthias Kunz, Katharina Lechner, Frank Meyer, Alexander Schmeißer, Saskia Meißler, Dörte Ahrens, Katja Neumann, Hendrik Mattern, Oliver Speck, Daniel Behme, Ildiko Rita Dunay, Ute Seeland, Stefanie Schreiber, Rüdiger Braun-Dullaeus

**Affiliations:** 1 Universitätsklinik für Kardiologie und Angiologie, Medizinische Fakultät, Otto-von-Guericke-Universität, Leipziger Str. 44, 39120 Magdeburg, Deutschland; 2 Deutsches Zentrum für Psychische Gesundheit (DZPG), Magdeburg, Deutschland; 3 Deutsches Zentrum für Neurodegenerative Erkrankungen (DZNE), Magdeburg, Deutschland; 4 Center for Intervention and Research on adaptive and maladaptive brain Circuits underlying mental health (C-I-R-C), Magdeburg, Deutschland; 5 Klinik für Herz- und Kreislauferkrankungen, Deutsches Herzzentrum München, Technische Universität, München, Deutschland; 6 DZHK (German Centre for Cardiovascular Research), partner site Munich Heart Alliance, München, Deutschland; 7 Universitätsklinik für Allgemein‑, Viszeral‑, Gefäß- und Transplantationschirurgie, Medizinische Fakultät, Otto-von-Guericke-Universität, Magdeburg, Deutschland; 8 Universitätsklinik für Neurologie, Medizinische Fakultät, Otto-von-Guericke-Universität, Magdeburg, Deutschland; 9 Biomedical Magnetic Resonance, Faculty of Natural Sciences, Otto-von-Guericke University, Magdeburg, Deutschland; 10 Center for Behavioral Brain Sciences (CBBS), Magdeburg, Deutschland; 11 Institut für Physik, Fakultät für Naturwissenschaften, Otto-von-Guericke- Universität, Magdeburg, Deutschland; 12 Universitätsklinik für Neuroradiologie, Otto-von-Guericke-Universität, Magdeburg, Deutschland; 13 Institut für Inflammation und Neurodegeneration, Otto-von-Guericke-Universität, Magdeburg, Deutschland; 14 Institut für Sozialmedizin, Epidemiologie und Gesundheitsökonomie, Charité – Universitätsmedizin, Berlin, Deutschland; 15 Zentrum für Innere Medizin, Sektion Geschlechtersensible Medizin und Prävention, Medizinische Fakultät, Otto-von-Guericke-Universität, Magdeburg, Deutschland

**Keywords:** Kardiovaskuläre Risikofaktoren, Lebensstilfaktoren, Demographischer Wandel, Geschlecht, Prävention, Cardiovascular risk factors, Lifestlye factors, Demographic change, Sex, Prevention

## Abstract

Kardiovaskuläre Risikofaktoren (Bluthochdruck, Rauchen, Übergewicht, Diabetes mellitus Typ 2, Dyslipidämie, körperliche Inaktivität) steigen mit zunehmendem Alter, insbesondere ab dem mittleren Erwachsenenalter, deutlich an, wobei Frauen wesentlich stärker betroffen sind. In der Bevölkerung Sachsen-Anhalts ist die Prävalenz kardiovaskulärer Risikofaktoren stark erhöht, und die Bevölkerungsstruktur in Sachsen-Anhalt ist besonders geprägt von einem hohen Durchschnittsalter sowie einer hohen Morbiditäts- und Mortalitätsrate aufgrund von kardiovaskulären Erkrankungen. Somit bietet Sachsen-Anhalt einen Modellcharakter für die demografische Entwicklung in Europa. Dieser Übersichtsbeitrag thematisiert Strategien zur Umsetzung zielgruppenspezifischer, kardiovaskulärer Präventionsstrategien im Bundesland Sachsen-Anhalt unter besonderer Berücksichtigung von Alter und Geschlecht. Indem präventivmedizinische Einrichtungen aufgebaut und innovative Versorgungsmöglichkeiten für kardiovaskuläre Risikopatienten geschaffen werden, soll Prävention auch dem ländlichen Bereich zugänglich gemacht werden.

## Abstract (en)

Cardiovascular risk factors (high blood pressure, smoking, overweight, type 2 diabetes, dyslipidemia, physical inactivity) substantially rise with increasing age, particularly after middle age, whereby women are affected to a much greater extent. In the population of Saxony-Anhalt the prevalence of cardiovascular risk factors is clearly increased and the population structure in Saxony-Anhalt is particularly characterized by a high average age as well as high morbidity and mortality rates due to cardiovascular diseases. Saxony-Anhalt therefore provides a model character for the demographic development in Europe. This review article discusses strategies for the implementation of target group-specific cardiovascular preventive strategies in the Federal State of Saxony-Anhalt with special consideration of age and sex. When preventive medicine facilities are established and innovative treatment possibilities for patients with cardiovascular risks are created, prevention should also become available in rural areas.

Dieser Übersichtsbeitrag thematisiert Strategien zur Umsetzung zielgruppenspezifischer, kardiovaskulärer Präventionsstrategien im Bundesland Sachsen-Anhalt unter besonderer Berücksichtigung von Alter und Geschlecht. Der Aufbau präventivmedizinischer Einrichtungen und innovativer Versorgungsmöglichkeiten von kardiovaskulären Risikopatienten soll diesen innovativen Ansatz der Prävention auch für den ländlichen Bereich zugänglich machen.

Die Lebenserwartung von Frauen (Platz 14/16) und Männern (Platz 15/16) ist in Deutschland geringer im Vergleich zu anderen westeuropäischen Ländern. Herz-Kreislauf-Erkrankungen sind die Todesursache Nummer 1 bei beiden Geschlechtern, die Verteilung der einzelnen kardiovaskulären Erkrankungen, die zum Tod geführt haben, ist aber unterschiedlich. Zum Beispiel ist die hypertensive Herzerkrankung, die mit Platz 8 der 10 häufigsten Ursachen bei Frauen gelistet ist, bei Männern nicht unter den ersten 10 Ursachen zu finden [21]. Daher erbringt eine Analyse von Studiendaten, getrennt nach dem biologischen Geschlecht, einen unabdingbaren Mehrwert, um personalisierte Präventionsstrategien zu entwickeln.

Die Bevölkerungsstruktur in Sachsen-Anhalt ist besonders geprägt von einem hohen Durchschnittsalter sowie einer hohen Morbiditäts- und Mortalitätsrate aufgrund von kardiovaskulären Erkrankungen [5]. Die Bevölkerung in Sachsen-Anhalt ist im bundesweiten Vergleich die älteste (2022: Bundesdurchschnitt: 44,6 Jahre/Sachsen-Anhalt: 47,9 Jahre) und weist eine deutlich erhöhte Prävalenz an kardiovaskulären Risikofaktoren (Bluthochdruck, Rauchen, Übergewicht, Diabetes mellitus Typ 2, Dyslipidämie, körperliche Inaktivität) auf (Abb. 1; Tab. 1). Somit bietet Sachsen-Anhalt einen Modellcharakter für die demografische Entwicklung in Europa.

**Abb. 1:**
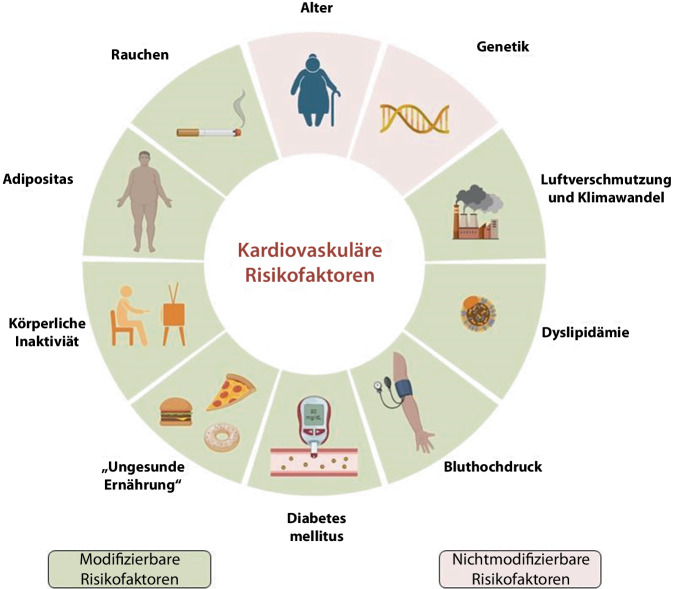
Kardiovaskuläre Risikofaktoren. (Erstellt mit Biorender)

**Tab. 1:** Prävalenz kardiovaskulärer Risikofaktoren in Sachsen-Anhalt vs. Gesamtdeutschland (mod. nach Diederichs et al. [7])

Risikofaktor	Prävalenz (%)			
	Sachsen-Anhalt		Deutschland	
	Männer	Frauen	Männer	Frauen
Diabetes mellitus	9,9	13,6	8,7	9,2
Hypertonie	42,5	38,4	32,5	31,1
Rauchen	34,5	25,1	32,6	24,9
Adipositas	17,6	23,2	16,3	15,7
Körperliche Inaktivität	44,1	40,8	35,4	35,3

Kardiovaskuläre Risikofaktoren steigen mit zunehmendem Alter, insbesondere ab dem mittleren Erwachsenenalter, deutlich an, wobei Frauen wesentlich stärker betroffen sind. Dies führt im Verlauf zu einer Beeinträchtigung der Lebensqualität und Autonomie im Alltag und hat für das Gesundheitssystem extreme infrastrukturelle und finanzielle Belastungen zur Folge.

## Prävalenz kardio- und neurovaskulärer Erkrankungen

Wie aktuelle Daten der „BURDEN-2020-Studie“ zeigen, ist Sachsen-Anhalt im bundesweiten Vergleich Spitzenreiter bei den Häufigkeiten von koronarer Herzkrankheit (KHK), Herzinsuffizienz, Schlaganfall und Demenz (Tab. 2). Das interaktive Projekt „Krankheitslage Deutschland“ (www.krankheitslage-deutschland.de) stellt eine Übersicht zu ausgewählten Krankheiten aller Einwohner in den Regionen Deutschlands zur Verfügung. Die gesteigerte Prävalenz kardio- und neurovaskulärer Erkrankungen resultiert in einer deutlich erhöhten Mortalitätsrate, Morbidität und Krankheitslast in Sachsen-Anhalt [18].

**Tab. 2:** Prävalenz kardio- und neurovaskulärer Erkrankungen in Sachsen-Anhalt im Vergleich zum Bundesdurchschnitt (altersstandardisiert; mod. nach Robert Koch-Institut [20])

Erkrankung	Prävalenz (%)					
	Sachsen-Anhalt			Deutschland		
	Gesamt	Männer	Frauen	Gesamt	Männer	Frauen
Koronare Herzkrankheit (KHK)	7,0 (4,8–10,6)	9,1 (5,1–15,6)	5,3 (3,3–8,3)	5,8 (5,4–6,3)	6,6 (5,9–7,4)	5,1 (4,5–5,7)
Schlaganfall	1,7 (0,8–3,8)	2,0 (0,5–6,3)	1,7 (0,7–4,1)	2,3 (2,0–2,6)	2,3 (1,9–2,8)	2,1 (1,7–2,6)
Depressive Symptomatik	9,1 (5,9–13,9)	7,7 (3,9–14,6)	10,6 (5,9–18,3)	8,3 (7,7–9,0)	7,5 (6,7–8,5)	8,8 (8,0–9,7)

Im Rahmen der Erhebung wurde die KHK definiert als Z. n. Myokardinfarkt bzw. KHK mit Angina-pectoris-Symptomatik (APS)/chronischen Beschwerden

Es existieren modifizierbare Risikofaktoren für Herz-Kreislauf-Erkrankungen und die Gesamtmortalität

Herz- und Kreislauferkrankungen sowie neurovaskuläre Erkrankungen verursachen in Deutschland nicht nur die meisten Todesfälle, sondern auch deutschlandweit 13 % aller Gesundheitskosten (56,7 von 431,8 Mrd. €; Statistisches Bundesamt 2023). Das „Global Cardiovascular Risk Consortium“ analysierte an einer globalen Kohorte von über 1,5 Mio. Teilnehmenden mit einem Anteil von 54 % Frauen die Wirkung von 5 modifizierbaren Risikofaktoren (Body-Mass-Index [BMI], systolischer Blutdruck, Low-Density-Lipoproteins[LDL]-Cholesterin, Rauchen, Diabetes) auf die Inzidenz von Herz- und Kreislauferkrankungen und die Gesamtmortalität. Frauen hatten insgesamt eine niedrigere Ereignisrate als Männer. Die Daten zeigen, dass der Einfluss der 5 Risikofaktoren bei Frauen (attributables Risiko 57,2 %) größer ist als bei Männern (52,6 %) [13].

## Ansätze zur kardiovaskulären Prävention

Der Aufbau interdisziplinärer Präventionsprogramme sowie der Auf- und Ausbau medizinischer Forschungs- und Versorgungsstrukturen (mit einem spezifischen Fokus auf *Präventionsmedizin*) zur Durchführung einer demografischen, kardiovaskulären Prävention in Sachsen-Anhalt setzt die Initiierung einer umfangreichen und konsentierten gesundheitspolitischen Strategie voraus. Anknüpfungspunkt bieten auch die bereits existierenden Leitlinien zur vaskulären Prävention (z. B. DEGAM-Leitlinie „Hausärztliche Risikoberatung zur kardiovaskularen Prävention“ [4], DGK-Leitlinie „Prävention von Herz- und Kreislauferkrankungen“ [8], DGN-Leitlinie „Sekundärprophylaxe ischämischer Schlaganfall und transitorische ischämische Attacke“ [6]). Perspektivisch bieten telemedizinische Präventionsansätze eine Möglichkeit zur Verbesserung der Umsetzung der flächendeckenden leitlinienbasierten Prävention.

### Interdisziplinäre Programme

Die Entwicklung, Implementierung und Umsetzung kardiovaskulärer Präventionsprogramme erfordern die interdisziplinäre Expertise aus geschlechtersensibler Medizin, Ernährungswissenschaft, Physiotherapie, Sportwissenschaft, Psychologie, Soziologie, Public Health und zahlreichen weiteren Disziplinen.

Gemeinsame intersektorale Initiativen von Akteuren der Politik, Krankenkassen, Medizin (ambulant und stationär), Bildungssysteme (Kindergarten, Schule, Universitäten) und Zivilgesellschaften (z. B. Sportvereine, Patienten-Selbsthilfegruppen) sind diesbezüglich unverzichtbar.

Die Projekte müssen langfristig konzipiert und durch gesicherte Finanzierung verstetigt werden

Projekte wie die „Initiative Herzgesundheit in Sachsen-Anhalt“ können als Ansatzpunkte genutzt werden (https://www.dein-herz-und-du.de/initiative-herzgesundheit). Dort wird einmal jährlich eine Aktionswoche zur Herzgesundheit mit vielfältigen Veranstaltungen (z. B. Screeninguntersuchungen zu Bluthochdruck und Dyslipidämie, Informationsveranstaltungen, Podiumsdiskussionen) zu Prävention und Therapie von Herz- und Kreislauferkrankungen organisiert. Diese Projekte müssen langfristig konzipiert und durch gesicherte Finanzierung verstetigt werden.

### Aufbau medizinischer Forschungs- und Versorgungsstrukturen

In der medizinischen Forschungs- und Versorgungsrealität ist die Thematik der Prävention dramatisch unterrepräsentiert. Im Jahr 2021 wurden lediglich 6,6 % der Gesundheitsausgaben für Prävention ausgegeben. Im Studium der Humanmedizin und der Weiterbildung sind eine Ausweitung und Verstetigung von Lehrinhalten zur Prävention zwingend erforderlich. Ein „Best-practice“-Beispiel ist der DGK-Sachkundekurs „Spezielle kardiovaskuläre Prävention“ [24].

Die Entwicklung und Umsetzung personalisierter Präventions- und Interventionsangebote erfordern darüber hinaus eine enge interdisziplinäre Zusammenarbeit von niedergelassenen Hausärzten, Fachärzten und Kliniken zahlreicher Fachrichtungen (Pädiatrie, Kardiologie, Angiologie, Diabetologie, Endokrinologie, Neurologie, Immunologie, Rheumatologie, Psychiatrie, Gefäßchirurgie, Sportmedizin, Physiotherapie, Psychologie/Psychosomatik etc.).

Das Zentrum für Gefäßgesundheit in Magdeburg betreibt interprofessionelle kardiovaskuläre Prävention

Realisiert wird diese Infrastruktur zur interprofessionellen Zusammenarbeit durch den Aufbau einer interdisziplinären Forschungs- und Versorgungsstruktur zur kardiovaskulären Prävention an der Otto-von-Guericke-Universität zu Magdeburg. Dieses Zentrum für Gefäßgesundheit unter der Initiative von Herrn Prof. Dr. med. Rüdiger Braun-Dullaeus (Universitätsklinik für Kardiologie und Angiologie) und Frau Prof. Dr. med. Stefanie Schreiber (Universitätsklinik für Neurologie) verfolgt die folgenden Inhalte und Ziele:

- eine interdisziplinäre Translationsforschung,
- eine Stärkung der vaskulären Prävention in Sachsen-Anhalt,
- die Entwicklung von innovativen und personalisierten Interventions- und Versorgungsangeboten (z. B. digitale kardiovaskuläre Präventionsangebote).

Mit der neu implementierten Professur für geschlechtersensible Medizin (Frau Prof. Dr. med. Ute Seeland) wird zudem der Bereich der personalisierten geschlechtersensiblen Prävention und Medizin systematisch entwickelt und erforscht.

Dazu werden u. a. modernste Methoden der nichtinvasiven Bildgebung mithilfe der Hochfeld-Magnetresonanztomographie (Hochfeld-MRT) eingesetzt, um pathophysiologische Mechanismen zu verstehen. Eine Besonderheit am Standort Magdeburg ist das neue 7‑Tesla-Connectome-MRT. Dieses in Europa einmalige Gerät verbessert die Darstellung von mikrostrukturellen Veränderungen, um beispielsweise pathophysiologische Mechanismen frühzeitig erkennen zu können.

Ein weiterer besonderer und in Ostdeutschland bisher einzigartiger Ansatz ist die enge Verzahnung von Neurowissenschaft, Immunologie und Kardiologie. Hintergrund sind die gemeinsamen Risikofaktoren für kardiovaskuläre und neurologische Erkrankungen und potenziell pathophysiologische Mechanismen. Exemplarisch steht hier die mikrovaskuläre Dysfunktion, ein potenzieller Mechanismus, der zur Herzinsuffizienz mit erhaltener Pumpfunktion („heart failure with preserved ejection fraction“, HFpEF) sowie zur zerebralen Mikroangiopathie beitragen kann.

### Strategien über die gesamte Lebensspanne

**Abb. 2:**
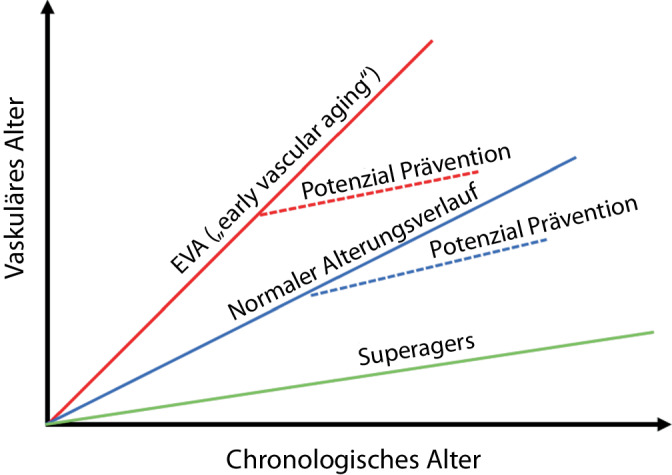
Gegenüberstellung von Personen mit einem beschleunigten vaskulären Alterungsverlauf (*EVA [„early vascular aging“]*; insbesondere aufgrund genetischer Faktoren in Kombination mit einem erhöhten kardiovaskulären Risikoprofil), Personen mit einem *normalen Alterungsverlauf* und *Superagers* (Personen mit protektiven genetischen Faktoren und niedrigem kardiovaskulären Risikoprofil). Mit Zunahme des vaskulären Alters erhöht sich das Risiko für kardio- und neurovaskuläre Folgeerkrankungen. Durch frühzeitige Präventionsmaßnahmen kann der vaskuläre Alterungsprozess positiv beeinflusst werden

Kardio- und neurovaskuläre Erkrankungen sind ein Resultat vaskulärer Alterungsprozesse über die gesamte Lebensspanne, wobei die Geschwindigkeit der Gefäßalterung durch das Zusammenspiel von genetischen und von Lebensstilrisikofaktoren bestimmt wird. Ein „ungesunder Lebensstil“ übersetzt sich häufig in metabolisch-vaskuläre Risikofaktoren wie beispielsweise Diabetes mellitus Typ 2 und Adipositas und beschleunigt über eine Vielzahl von Mechanismen den vaskulären Alterungsprozess, was in einer früheren Manifestation von mikro- und makrovaskulären Endorganschäden resultiert. Durch frühzeitige Präventionsmaßnahmen kann der vaskuläre Alterungsprozess positiv beeinflusst werden (Abb. 2). Für die geschlechtersensible Medizin sind die Erkenntnisse zu den Effektstärken der einzelnen Risikofaktoren- oder -modifikatoren wichtig, um Präventionsstrategien für Geschlecht und Alter zu optimieren. Diabetes mellitus Typ 2 hat z. B. eine größere Effektstärke bezüglich des kardiovaskulären Risikos bei Frauen im Vergleich zu Männern [19]. Aus geschlechtersensibler Sicht spielen die Datenanalysen zu soziokulturellen Einflussfaktoren eine wichtige Rolle bei der Erforschung der Zeitfenster während einer Lebensspanne, die sich besonders für einen präventiven Ansatz eignen. Der interdisziplinäre Ansatz des Gefäßzentrums stellt hier einen besonderen Vorteil dar.

Im internationalen Vergleich wird deutlich, dass bereits 2010 die „American Heart Association“ (AHA) ein neues Konzept zur kardiovaskulären Gesundheit definierte, mit Fokus auf die Gesundheitsförderung und Prävention über die gesamte Lebensspanne und nicht auf die Behandlung von Krankheiten [12]. Das Kernelement des Konzepts besteht in der Vorstellung, dass Gesundheit mehr ist als die reine Abwesenheit von Krankheiten. Grundlegende Faktoren – als „life’s essential 8“ bezeichnet – sind (Tab. 3):

- gesunde Ernährung (insbesondere „Dietary Approaches to Stop Hypertension“ [DASH-Diät] und mediterrane Ernährung),
- Rauchverzicht,
- Schlafqualität und -quantität,
- Lipidmanagement (individuelle Zielwerte, basierend auf kardiovaskulärem Risiko),
- BMI,
- körperliche Aktivität,
- Blutdruckoptimierung (und)
- Blutzuckereinstellung.

**Tab. 3:** Komponenten des Lifestyle-8-CVH-Scores [12]

Lebensstilfaktor	Quantifizierung des CVH-Scores
Ernährung (Mediterranean Eating Pattern for Americans[MEPA]-Fragebogen, Punkte)	100: 15–16 80: 12–14 50; 8–11 25: 4–7 0: 0–3
Körperliche Aktivität (moderate Aktivität/Woche, min)	100: ≥ 150 90: 120–149 80: 90–119 60: 60–89 40: 30–59 20: 1–29 0: 0
Nikotinkonsum	100: niemals geraucht 75: Exnikotinabusus (vor > 5 Jahren) 50: Exnikotinabusus (vor 1–5 Jahren) 25: Exnikotinabusus (im letzten Jahr) 0: Raucher
Schlafdauer (h)	100: 7–< 9 90: 9–< 10 70: 6–< 7 40: 5–< 6 oder ≥ 10 20: 4–< 5 0: < 4
Body-Mass-Index (kg/m^2^)	100: < 25 70: 25,0–29,9 30: 30,0–34,9 15: 35,0–39,9 0: ≥ 40,0
Non-HDL-Cholesterin (mg/dl)^a^	100: < 130 60: 130–159 40: 160–189 20: 190–219 0: ≥ 220
Blutzucker (HbA_1c_)	100: Diabetes (HbA_1c_ <5,7) 60: Prädiabetes (HbA_1c_ 5,7–6,4) 40: Diabetes mit HbA_1c_ <7,0 30: Diabetes mit HbA_1c_ 7,0–7,9 20: Diabetes mit HbA_1c_ 8,0–8,9 10: Diabetes mit HbA_1c_ 9,0–9,9 0: Diabetes mit HbA_1c_ ≥10,0
Blutdruck (mm Hg)^b^	100: <120/<80 (optimal) 75: 120–129/<80 50: 130–139 oder 80–89 25: 140–159 oder 90–99 0: ≥160 oder ≥100

Der kardiovaskuläre Gesamtscore errechnet sich als arithmetisches Mittel der Einzelwerte

*CVH* „cardiovascular health“ (kardiovaskuläre Gesundheit), *HDL* High Density Lipoproteins

^a^ Bei Therapie: 20 mg zusätzlich addieren

^b^ Bei Therapie: 20 mm Hg addieren

Die „life’s essential 8“ bieten einen Ansatz zur Objektivierung des Risikoprofils über ein Scoring-System modifizierbarer Risikofaktoren mit Bildung eines individuellen kardiovaskulären Gesundheitsscores (CVH score, 0–100, Tab. 3). Ein hoher CVH-Score ist mit einer geringeren Prävalenz für Atherosklerose, Vorhofflimmern, Herzinsuffizienz, kognitive Beeinträchtigung und Depression assoziiert [1]. Frauen haben häufiger einen höheren Score als Männer [22]; ein Beispiel, dass auch diese CVH-Score-Berechnung getrennt nach dem Geschlecht unter Berücksichtigung der angepassten Normwerte erfolgen muss. In Deutschland existiert jedoch bisher kein digitaler bzw. automatisierter Kalkulator des CVH-Scores.

Frühzeitige Präventionsmaßnahmen können den vaskulären Alterungsprozess positiv beeinflussen

In Deutschland verbreiteter ist der SCORE‑2 zur Berechnung des individuellen Risikos innerhalb der nächsten 10 Jahre, ein kardiovaskuläres Ereignis zu erleiden. Elemente des SCORE‑2 sind:

- Alter,
- Non-HDL-Cholesterin,
- systolischer Blutdruck,
- Nikotinkonsum,
- Geschlecht.

Diesbezüglich existieren im Internet automatische Risikokalkulatoren zur Berechnung (z. B. http://www.scores.bnk.de/score2--2021-.html; ESC CVD Risk Calculation App). Basierend auf dem individuellen Risikoprofil und dem Vorliegen von Krankheiten kann Prävention unterteilt werden in:

- *primordiale Prävention* (Gesundheitsförderung durch die Eliminierung oder Vorbeugung von Risikofaktoren),
- *Primärprävention* (Verhinderung von Erkrankungen durch Einstellung von bestehenden Risikofaktoren) und
- *Sekundärprävention *(Verhinderung von Krankheitsprogression und Rezidiven).

Die Effektstärken von Maßnahmen der Prävention zur Verlangsamung des vaskulären Alterungsprozesses nehmen über die Lebensspanne ab, da das Gesamtrisiko ganz maßgeblich von der Expositionsdauer gegenüber einem gegebenen Risikofaktor über die Zeit bestimmt wird. Deswegen ist es unerlässlich, Präventionsstrategien, die bereits im Kindesalter ansetzen, zu entwickeln.

Kernelemente im Kindes- und Jugendbereich sind:

- gesundes Schulessen (z. B. kostenloses Schulessen für alle Kinder und Jugendliche, um bereits früh das Bewusstsein für eine ausgewogene Ernährung zu schaffen),
- körperliche Aktivität (Schulsport, Vereinssport) sowie
- Aufklärung über einen gesunden Lebensstil (insbesondere Rauchverzicht).

### Zielgruppenspezifische Strategien

Die Wirksamkeit und Kosteneffektivität von Präventionsansätzen über die gesamte Lebensspanne ist nachgewiesen [2, 3, 23, 26]. Initial erfordert eine effektive Präventionsstrategie eine finanzielle und infrastrukturelle Investition. Eine Reduktion von Folgeerkrankungen und Kosten im Gesundheitssystem ist erst mittel- bis langfristig zu erwarten. Das Potenzial für die Reduktion von Folgeerkrankungen und Einsparungen im Gesundheitssystem ist dennoch enorm.

Positive Effekte von Präventionsansätzen sind bei Patienten mit einem hohen kardiovaskulären Risikoprofil (z. B. Bluthochdruckpatienten mit metabolischem Syndrom) früher nachzuweisen als bei Kindern und Jugendlichen. Die arterielle Hypertonie ist der bedeutendste modifizierbare Risikofaktor für kardiovaskuläre (z. B. Herzinfarkt, Herzinsuffizienz), neurovaskuläre (z. B. Schlaganfall) und demenzielle Erkrankungen (insbesondere vaskuläre Demenzen) [17]. Die Hypertonie ist ein stärkerer Risikofaktor für kardiovaskuläre Erkrankungen bei Frauen im Vergleich zu Männern und beginnt schon bei niedrigeren Blutdruckwerten [9].

Arterielle Hypertonie ist der wichtigste modifizierbare Risikofaktor für kardiovaskuläre Erkrankungen

Bluthochdruck ist laut der aktuellen Leitlinie der European Society of Cardiology (ESC) aus dem Jahr 2024 anhand des Vorliegens von reproduzierbar gemessenen Ruheblutdruckwerten in der Praxis/Klinik ≥ 140 mm Hg systolisch und/oder ≥ 90 mm Hg diastolisch definiert [14] (Tab. 4). Systolische Blutdruckwerte von 120–139 mmHg und diastolische Blutdruckwerte von 70–89 mmHg werden als erhöhte Blutdruckwerte eingestuft. Die aktuelle Leitlinie betont stärker die individuelle Patientenzentrierung, Kombinationstherapien, Lebensstiländerungen und den Einsatz digitaler Technologien im Vergleich zur vorherigen Leitlinie aus dem Jahr 2018. Um eine genaue Diagnose und Überwachung zu gewährleisten wird auf die präzise und regelmäßige Messung des Blutdrucks, einschließlich Heim- und ambulanten Blutdruckmonitorings, hingewiesen. Die Empfehlungen zur Behandlung von Hypertonie bei Frauen (einschließlich Schwangerschaft) wurden aktualisiert.

**Tab. 4:** Einteilung der arteriellen Hypertonie in Schweregrade (abhängig von den in der Praxis/Klinik gemessenen Blutdruckwerten in Ruhe) [14]

Kategorie	Systolisch/diastolisch (mm Hg)
Nicht erhöhte Blutdruckwerte	< 120 und < 70
Erhöhte Blutdruckwerte	120–139 und/oder 70–89
Hypertonie Grad I	140–159 und/oder 90–99
Hypertonie Grad II	160–179 und/oder 100–109
Hypertonie Grad III	≥ 180 und/oder ≥ 110
Isolierte systolische Hypertonie	≥ 140 und < 90

Die Therapie basiert auf Lebensstilmodifikation (u. a. Gewichtsreduktion, körperliche Aktivität) sowie einer personalisierten Medikation (Antihypertensiva) [11, 15]. Der prognostische Nutzen der antihypertensiven Therapie im Hinblick auf Lebensqualität, Morbidität und Mortalität wurde in zahlreichen randomisierten, kontrollierten Interventionsstudien belegt. Eine Blutdruckeinstellung gemäß den empfohlenen Zielwerten kann das Risiko für kardio- und neurovaskuläre Erkrankungen um 30 % senken [16]. Dennoch erreicht die Hälfte der Bluthochdruckpatienten nicht die von den Fachgesellschaften empfohlenen individuellen Zielwerte.

Die Hypertonieprävalenz in Sachsen-Anhalt übersteigt mit 41 % den Bundesdurchschnitt (32 %) signifikant, mit dringender Notwendigkeit für wirksame flächendeckende Versorgungskonzepte [7].

Das Bluthochdruckrisiko steigt ab dem mittleren Erwachsenenalter deutlich an, sodass im Rahmen des demografischen Wandels mit einer weiteren Zunahme der bundes- und landesweiten Hypertonieprävalenz auf über 50 % im Jahr 2050 zu rechnen ist. Ab dem Seniorenalter (> 70 Jahre) ist die Prävalenz der Hypertonie bei Frauen häufiger als bei Männern (Statistisches Bundesamt, 2021); dies gilt insbesondere für systolisch betonte Hypertonieformen.

Aktuell betragen die jährlichen Kosten für die Folgen der arteriellen Hypertonie ca. 10 % der gesamten Gesundheitsausgaben; für Sachsen-Anhalt entspricht das mindestens 1 Mrd. €/Jahr. Bis zum Jahr 2050 ist mit einer Zunahme der Gesundheitsausgaben um (*beachte*) 300 % zu rechnen. Beispielhaft ist als Folgeerkrankung die Herzinsuffizienz genannt als häufigster Grund für wiederkehrende Krankenhausaufenthalte in Deutschland. In Sachsen-Anhalt betragen die aktuellen jährlichen Gesundheitskosten für Herzinsuffizienz ca. 4 Mrd. € (bei 110.000 Patienten). In Sachsen-Anhalt könnten mittel- bis langfristig jährlich 800 Mio. € eingespart werden, würden nur 20 % der Herzinsuffizienzerkrankungen durch eine leitliniengerechte Bluthochdrucktherapie verhindert werden.

### Digitale kardiovaskuläre Prävention

Einen potenziell vielversprechenden Ansatz zur kardiovaskulären Prävention bietet die Telemedizin. Insbesondere im Flächenland Sachsen-Anhalt und der aktuellen Situation des Hausärztemangels könnte via Telemedizin die Patientenversorgung optimiert werden. Im Bereich der Herzinsuffizienz ist die telemedizinische Patientenversorgung neuerdings die Regelversorgung (*G‑BA-Beschluss, BAnz AT 04.03.2021 B1, 2022*).

Für die arterielle Hypertonie hat die japanische HERB-DH1-Studie gezeigt, dass eine appbasierte digitale Hypertonietherapie einer konventionellen Therapie bezüglich

- des Erreichens der Zielwerte,
- der Optimierung der Lebensstilfaktoren sowie
- der Prävention von kardio- und neurovaskulären Folgeerkrankungen signifikant überlegen ist [10].

In einem aktuellen Forschungsprojekt wird im nördlichen Sachsen-Anhalt die Effektivität einer *Di*gitalen *K*ardiovaskulären *P*rävention (DIKAP) bei Patienten mit Bluthochdruck und erhöhtem kardiovaskulären Risikoprofil untersucht.

Elemente der DIKAP sind:

- digitale Informationsmaterialien zur arteriellen Hypertonie (Patientenschulungen, narrative Visualisierung von Risikofaktoren und Folgeerkrankungen, Lebensstilberatung, Unterstützung des „self-empowerment“),
- telemedizinisches Monitoring der kardiovaskulären Parameter (z. B. Blutdruck, Körpergewicht, Medikamentenplan),
- telemedizinische Konsultationen durch einen Arzt bzw. kardiovaskulären Präventionsassistenten (Optimierung der medikamentösen und nichtmedikamentösen Therapie) sowie
- auf künstlicher Intelligenz (KI) basierte Datenanalyse („big data“). Die KI-basierte Datenanalyse könnte in diesem Rahmen Risikopatienten identifizieren und Präventionsprogramme optimieren.

## Schlussfolgerung und Perspektive

Im Kontext des demografischen Wandels und der daraus resultierenden potenziell dramatischen Konsequenzen für das Gesundheitssystem ist eine Strategie zur Förderung der vaskulären Prävention zwingend notwendig.

In diesem Zusammenhang soll angemerkt werden, dass es sowohl am Universitätsklinikum Magdeburg als auch am Universitätsklinikum Halle (Saale) keine eigenständige Institution für Präventions- bzw. Sportmedizin gibt. Eine selbstständige Struktureinheit zur Förderung und zur Stärkung der vaskulären Prävention ist jedoch im Bundesland Sachsen-Anhalt zwingend erforderlich. Gute Beispiele sind das Institut für Präventive Sportmedizin und Sportkardiologie (Klinikum rechts der Isar, Technische Universität München) und die Sektion Sport- und Rehabilitationsmedizin des Universitätsklinikums Ulm.

Im Rahmen des aktuell im Aufbau befindlichen „Zentrum für Gefäßgesundheit“ soll die vaskuläre Prävention in Sachsen-Anhalt gestärkt werden. Das Ziel ist eine engere Kooperation mit lokalen Gesundheitsakteuren (Krankenkassen, Gesundheitspolitik, niedergelassene Ärzte etc.).

Für Patienten mit spezifischem Wunsch und/oder klinischer Notwendigkeit wird bereits jetzt eine Anlaufstelle in der interdisziplinären Hochschulambulanz für Prävention (mdzg@med.ovgu.de; Ansprechpartner Dr. Patrick Müller) geboten. Für Patientinnen und Patienten steht zudem ab Ende 2024 die erste Hochschulambulanz für geschlechtersensible Medizin und Prävention zur Verfügung.

Im Rahmen des Zentrum für Gefäßgesundheit ist zusätzlich der Aufbau einer überregionalen Kohorte zur Untersuchung von vaskulären Alterungsprozessen geplant. Diesbezüglich erhalten die Probanden

- eine umfangreiche sportmedizinische (u. a. Spiroergometrie, Testung der Muskelkraft) und vaskuläre Diagnostik (u. a. Ultraschall des Herzens und der Gefäße, Messung der Gefäßsteifigkeit),
- eine neuropsychologische Diagnostik,
- umfangreiche Biomarkerbestimmungen (und)
- ein MRT von Gehirn und Herz.

Über die Zuweisung von interessierten Probanden und Patienten sowie die Förderung des Präventions- und Forschungsansatzes würden wir uns freuen.

## Fazit für die Praxis

- Die Bevölkerung Sachsen-Anhalts ist im bundesweiten Vergleich die älteste und weist eine deutlich erhöhte Prävalenz kardiovaskulärer Risikofaktoren auf. Sachsen-Anhalt bietet damit einen Modellcharakter für die demografische Entwicklung in Europa.
- Kardiovaskuläre Risikofaktoren steigen mit zunehmendem Alter deutlich an; Frauen sind wesentlich stärker betroffen. Dies führt im Verlauf zur Beeinträchtigung der Lebensqualität und Autonomie im Alltag und hat für das Gesundheitssystem extreme infrastrukturelle und finanzielle Belastungen zur Folge.
- Kardio- und neurovaskuläre Erkrankungen sind ein Resultat vaskulärer Alterungsprozesse über die gesamte Lebensspanne; die Geschwindigkeit der Gefäßalterung wird durch das Zusammenspiel von genetischen und von Lebensstilrisikofaktoren bestimmt. Frühzeitige Präventionsmaßnahmen können den vaskulären Alterungsprozess positiv beeinflussen.
- Gemeinsam mit zahlreichen Akteuren wollen wir in Sachsen-Anhalt ein „Leuchtturmprojekt“ zur kardiovaskulären Prävention aufbauen und einen Beitrag zur erfolgreichen Bewältigung des demografischen Wandels leisten.
